# The Greek sovereign debt crisis as an important chapter in the history of the European Monetary Union: empirical evidence and some thoughts on implications for investors and financial risk managers

**DOI:** 10.1007/s12297-022-00529-0

**Published:** 2022-09-05

**Authors:** Johannes Tholl, Christoph Schwarzbach

**Affiliations:** 1 Universität Bayreuth, Universitätsstraße 30, 95447 Bayreuth, Germany; 2 Institute of Information Systems Research, Gottfried Wilhelm Leibniz University Hannover, Koenigsworther Platz 1, 30167 Hannover, Germany

**Keywords:** European sovereign debt crisis, Risk premia, Government bonds, Greece, E43, G15, H63

## Abstract

After the burst of the US real estate price bubble in 2008, the events in Greece played, at least for some time, a unique role on the path towards the European sovereign debt crisis. We examine this issue empirically and then discuss some relevant questions for policymakers, regulators, fixed income investors, and financial risk managers. Our findings should be of interest when modeling risk premia determined in the market for sovereign bonds issued by countries that have introduced the Euro. Given the exposure of the European insurance industry to these fixed-income securities, the results are important for asset managers and financial risk managers that are working in this segment of the financial services industry.

## Abstract (de)

Nach dem Platzen der Immobilienpreisblase in den USA haben die Vorgänge in Griechenland eine hohe Bedeutung für die Entwicklungen gehabt, die dann letztlich zu einer Staatsschuldenkrise in Europa geführt haben. Wir analysieren die Zusammenhänge empirisch und diskutieren dann in diesem Kontext relevante Fragestellungen für Wirtschafts- und Finanzpolitiker, Regulierungsbehörden, Investoren und Risikomanager in der Finanzbranche. Unsere Ergebnisse dürften von besonderer Bedeutung für die Modellierung von Risikoprämien am Markt für Staatsanleihen aus der Eurozone sein. Mit Blick auf das Anlageverhalten von Versicherungsunternehmen haben die Ergebnisse unserer empirischen Untersuchungen sicherlich eine hohe Relevanz für Kapitalanleger und Risikomanager in dieser Branche.

## Introduction

The European sovereign debt crisis undoubtedly had a significant impact on international financial markets (see, for instance, Gruppe and Lange 2014 and Wegener et al. 2016a). Prior to this, government bond yields that European Monetary Union (EMU) member countries had to pay to their investors were closely related to each other. In fact, at the beginning of the European common currency, there was interest rate convergence among short-, medium- and long-term yields of government bonds issued by EMU member countries. This was no surprise since there is only one Main Refinancing Operations Announcement Rate in the EMU that is determined by the European Central Bank (ECB) and since the advent of the common currency, the exchange rate risk was eliminated (see, for example, Wegener et al. 2016b and Gruppe et al. 2017). Moreover, there were no major fears about fiscal problems in the EMU member states in the early days of the currency union and government bonds issued by countries that had introduced the Euro were considered to be risk-free assets until the Greek debt swap (see, most importantly, Gruppe and Lange 2014 and Sibbertsen, Wegener and Basse 2014). Meanwhile, fears about sovereign credit risk and possibly even redenomination risk increased the risk premia that at least some of the less fiscally stable member countries had to provide to attract investors (see, amongst others, Basse 2014 and Sensoy et al. 2019). In fact, there seems to be a belief that risk premia before the crisis were too low and that there was an underpricing of sovereign credit risk in the EMU in this period (see, for example, Gibson et al. 2014 and Basse et al. 2018). Monetary policy is also relevant in this context (see, for instance, Eser and Schwaab 2016 and Afonso et al. 2018). The ECB helped to ease the funding problems of the less fiscally stable EMU governments. In sum, there are at least two pricing regimes for EMU government bonds—and most likely even more. These pricing regimes are characterized by significant differences between the risk premia demanded by buyers of sovereign debt issued by member states of the EMU that are considered to be fiscally stable and those considered to be more unstable.

In this context, it is important to note that Lempérière et al. (2017) stressed that there is still no clear picture of how financial markets determine risk premia. Consequently, a need for more empirical research seems to prevail. Most importantly, Gunay (2020) recently suggested using Granger causality tests (see, most importantly, Granger 1969) to improve our understanding of the information flows between different risk premia. We follow his example and investigate how the risk premia in the market for Greek sovereign debt affected the markets for other EMU government bonds. This empirical evidence is of particular interest because Arghyrou and Kontonikas (2012) argued that the fiscal problems in Greece were of special importance for some time and, at least in this period, also had major consequences for ending the tendency towards an interest rate convergence in the EMU. Given that EMU government bonds and related assets are an important asset class for European insurance companies (see, amongst others, Ludwig 2014 and Tholl et al. 2021), the empirical evidence reported here certainly is highly relevant for this sector. This is especially true for asset and financial risk managers in the life insurance industry. In fact, the current low interest rate environment that is also a consequence of the recent crisis events seems to be a significant problem for European life insurance companies (see, for example, Basse et al. 2014 and Berdin and Gründl 2015). Moreover, the fact that the EMU government bond market started to price sovereign credit risk also led to some critical questions about regulatory issues in the European insurance industry—namely with regard to the way Solvency II handles investments in sovereign government bonds (see, most importantly, Basse et al. 2012 and Ludwig 2014). Some more recent papers stimulated these discussions (see, amongst others, Meier et al. 2020 and Basse 2020).

The paper is structured as follows. Sect. 2 briefly reviews the relevant literature. The third section examines the difficulties in Greece in some detail and also analyzes monetary policy issues that are relevant in this context. Sect. 4 discusses some methodological issues and introduces the data set examined here. The results of the empirical investigations are presented and analyzed in Sect. 5. The sixth section concludes.

## Literature review

Looking at the EMU from a historical perspective, it is open to question how many different phases it went through. Among others, Lane (2012) states that the history of the EMU is divided into three phases—before the sovereign debt crisis, during the crisis, and after the crisis when pressure on fiscally instable countries began to ease (see also, Arghyrou and Kontonikas 2012). Tholl et al. (2021) suggest that the outbreak of COVID-19 can complement the above-mentioned three phases in EMU history as the beginning of a fourth period. Before the EMU was implemented, optimistic expectations were that the common currency would foster the ability, especially of peripheric member states, to attract financial market investors more easily. This would be achieved thanks to the EMU’s proposition of fiscal solidity, which is formally expressed by the convergence criteria as part of the Maastricht treaty (see, for instance, Franzmeyer 1995 and Söllner 2000). The treaty also determined three steps as a pre-condition for creating the EMU, starting with free capital flows among the member states, followed by a stronger cooperation among the national central banks, and ending in the introduction of the Euro as the common currency. The convergence criteria, which are also called Maastricht criteria, aimed at binding the EMU member states to fiscal consolidation and forcing their national banks towards a solid monetary policy. Fiscal solidity means that a country that wants to become a member of the EMU should have a public debt of not more than 60% of its GDP and an annual fiscal deficit of not more than 3% of GDP. From a monetary perspective, the Maastricht criteria stipulate that during an observation period of one year, a candidate country’s key interest rate is not allowed to exceed the policy rate of the three most solid EMU member countries by more than 200 base points, for two years before joining the EMU the exchange rate needs to be in-between a fixed fluctuation margin, and for one year of observation the country’s inflation rate should not exceed the three lowest rates of EMU member countries by more than 1.5 percentage points (see, for example, Fatás and Mihov 2003 and Camba-Mendez and Lamo 2004). The Maastricht criteria were complemented by the adoption of the Stability and Growth pact (SGP) in 1997, which intended to strengthen the commitment of the EMU member states to conduct a policy of fiscal solidity. In the absence of a common fiscal policy, the contract intended to obligate the Eurozone members to keep fiscal discipline in order to tackle a moral hazard problem: as the instrument of exchange rate adjustments is not available, members of the Euro area might be tempted to pursue a less stringent fiscal policy—not only in times of crisis. Moreover, investors might lose their disciplining influence since EMU member states issue bonds in a common currency instead of separate national currencies (see, Eijffinger and Hoogduin 2012). The Maastricht criteria, as well as the SGP, were subject to widespread discussions (see, for example, Bibow 2013 and Savona 2015). On the one hand, they supposedly do not sufficiently reflect the necessities of a Keynesian demand-supporting fiscal policy in times of external shocks. On the other hand, the rules are questioned as nowadays nearly all EMU members do not fulfill the fiscal requirements set by the Maastricht criteria.

**Fig. 1:**
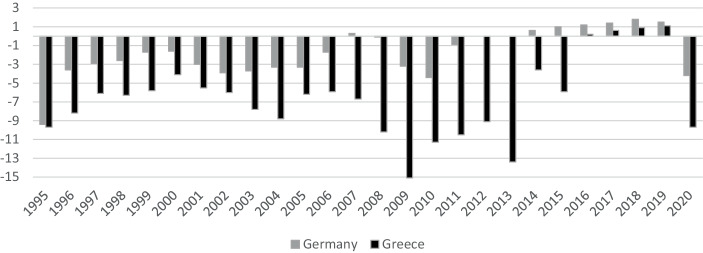
Government deficit/surplus in percentages of Gross Domestic Product. Source: Eurostat (2021a)

Before the start of the sovereign debt crisis, the expectations of converging EMU sovereign bond yields corresponded to later developments—independent of the maturity (see, for example, Gruppe et al. 2017; Feldstein 2015; Dellas and Tavlas 2012). This trend came to an end only because of the outbreak of the sovereign debt crisis. This second phase of the EMU is characterized by sharp increases in sovereign bond spreads (see, for example, Ludwig 2014). Greece’s financial situation provoked fears that it may be contagious and, therefore, could cause financial turmoil for other highly indebted EMU member states like Portugal, Ireland, Italy, and Spain. Additionally, France and Belgium experienced widening spreads (see, for example, de Santis 2012 and Claeys and Vašíček 2014). The contagion risk was also caused by the sovereign-bank-nexus that, among other things, already had contributed to the transition of the financial crisis to the sovereign debt crisis, which may have provoked a worsening of the financial crisis and thereby may have established a vicious circle (see, amongst others, Wegener et al. 2019 and Cuadros-Solas and Salvador Muñoz 2021). The idea of contagion is based on the assumption that restructuring Greek sovereign debt would also have a negative impact on banks that are creditors to Greece—whether as credit grantors or asset holders of Greek sovereign bonds (see, for example, Roman and Bilan 2012). These fears are supported by the fact that lendings to the home sovereign made up 11% of Greece’s banks’ assets. The Greek insurance and pensions fund sector’s exposure to its own government even amounted to 29% (see, Ardagna and Caselli 2014). To prevent a worsening of the financial crisis, the governments would be forced to provide financial rescue packages, which would aggravate the burden on the respective sovereign’s budget (see, for instance, de Santis 2012, Basse et al. 2012, and Fig. 1).

Despite the no-bail-out clause, in May 2010, the EMU members opted to provide bilateral credits to Greece, summing up to 80 billion EUR. This was accompanied by an IMF lending amounting to an additional 30 billion EUR, conditional on fiscal and structural reforms (see, Mink and de Haan 2013). Shortly after, the European Financial Stability Facility (EFSF) was created with a volume of 440 billion EUR to provide funds for EMU member states suffering financial turmoil (see, de Grauwe 2011; Gocaj and Meunier 2013). The funds were backed by financial guarantees of the EMU member states. At the same time, the IMF and the European Financial Stabilization Mechanism (EFSM) provided in total 310 billion EUR. This adds up the potentially available liquidity for states in financial need to 750 billion EUR. This framework was paralleled by the ECB’s announcement to purchase sovereign debt of EMU states. The EFSF was created to provide liquidity via loans to EMU countries at short notice (see, Horváth and Huizinga 2011) and was subject to criticism regarding its legal framework as an implicit step towards the communitization of public debt in the Eurozone. From a moral hazard perspective, this approach supposedly weakens a country’s incentive to follow a solid fiscal policy (see, Pisani-Ferry 2010).

On the monetary side, support was achieved by the Securities Market Programme (SMP) of the ECB, which aimed to stabilize the demand for sovereign bonds and bills coming under market pressure (see, e.g., Zettelmeyer et al. 2013). In July 2011, when it became clear that Greece would not be able to return to the financial markets in 2012, Eurozone member states further increased their financial support by 109 billion EUR in addition to 135 billion EUR contributed by the private sector (see, Zettelmeyer et al. 2013).

Due to the central banks’ actions against the financial crisis, there was little maneuvering space left for the monetary policy to counteract the arising sovereign debt crisis. Financial markets doubted that all members of the Eurozone would remain in the common currency area. These fears were only allayed with the famous Draghi speech on 26 July 2012 and the corresponding unconventional monetary policy instruments like asset purchase programs (e.g., OMT and LTRO) by the ECB (see, Acharya et al. 2019). They helped to provide liquidity to the financial markets in order to overcome a liquidity shortfall in the sovereign bond markets and, at the same time, stabilize the demand for sovereign bonds (see, Cour-Thimann and Winkler 2012). They also convinced financial markets that the EU institutions would do everything to maintain the EMU. The main arguments were, on the one hand, the volume of the ECB response and, on the other hand, the central bank’s promise to even strengthen its measures if they proved not to be sufficient. It marked the beginning of the third phase in EMU history—a time of recovery (see, Lane 2012).

Our empirical research (which will be reported later) provides strong evidence that Greece played an essential role in the sovereign debt crisis. This is due to its significant impact on the crisis development in other highly indebted EMU member states and the financial market’s reaction. Therefore, the history of the European currency area might be divided not only into three but rather into four phases. Without a doubt, there was a pre- and post-sovereign debt crisis phase in the EMU. Additionally, the phase in between could be divided into phases II a and II b of the EMU history. To understand the situation in Greece during the sovereign debt crisis, we look back at the main events and the issues discussed at the time as well as provide some background information.

## Greece’s special characteristics in the sovereign debt crisis

The until than severest crisis in EMU history started in October 2009, when Greece’s newly elected government announced that the forecast for its fiscal budget deficit of the same year would rise from 7% to 12% (see, Zettelmeyer et al. 2013). Considering an already elevated debt level and fiscal measures that fell short of expectations (see, Baum et al. 2016), in October 2009, Fitch decided to downgrade the Hellenic sovereign debt to A‑ and two months later, further to BBB. Standard & Poor’s and Moody’s, the two other relevant rating agencies, followed that development. This highlighted an issue that had been largely ignored by financial markets before, although the financial circumstances had already existed for some time (see, Akram et al. 2011). Six months later, due to the downgrades and the corresponding increased yield demands by investors, Greece was implicitly cut off from the financial markets and consequently about to run out of liquidity. The Greek government responded by adopting austerity measures aimed at preparing for bailout negotiations (see, Ardagna and Caselli 2014).

**Fig. 2:**
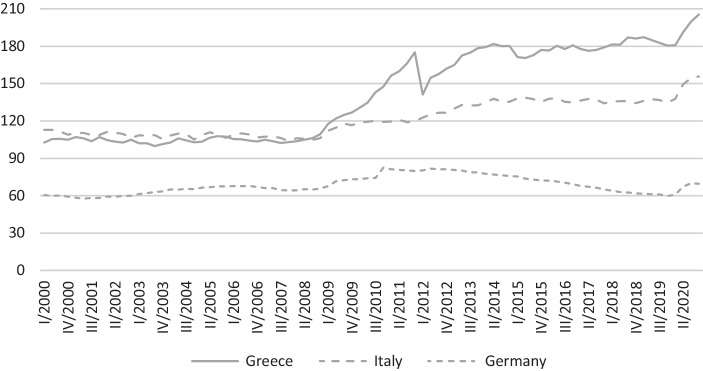
Public debt to GDP ratio in percentages. Source: Eurostat (2021b)

Greece’s worsening financial situation (see also, Fig. 2) fostered criticism that had already prevailed before but gained importance due to the crisis. Since its beginning but especially since the outbreak of the sovereign debt crisis, the EMU has faced criticism with regards to some basic ideas of economic theory: the idea of the “Optimum Currency Area” (OCA) by Mundell (1961), which was later enhanced by McKinnon (1963) (see, among others, Schwartz et al. 2013 as well as Jager and Hafner 2013, and the literature survey by Kunroo 2015) and the idea of public financial sustainability that is associated with a maximum debt level. According to the OCA theory, a region interconnected by trade but divided by national borders should aim to create a common monetary area as long as certain conditions are fulfilled. The latter should compensate the members for the disadvantage of abandoning their own monetary policy and the corresponding instruments. According to Mundell (1961), an OCA is given when asymmetric shocks can be absorbed. This can be achieved by facilitating a rapid adaptation of wage and price levels as well as high factor mobility which is to say not only capital but also labor mobility. The latter enables the workforce to move from regions with rising unemployment to regions capable of absorbing unemployed workers. The OCA theory claims that a country should maintain its own currency if the disadvantages of crisis adaptation mechanisms regarding price and wage changes outweigh the absence of a rapid crisis mechanism: exchange rate depreciation.

With regard to the EMU, there are a lot of relevant papers examining which countries should be part of this monetary union (see, for instance, Artis 2003 and Kim and Chow 2003). In summary, it can be stated that there does not seem to be a clear picture trying to answer this question. Savona (2015), for instance, has argued that the EMU might be a non-optimal currency area. It is open to discussion whether an OCA requires a common fiscal transfer system that compensates for a sudden decline in aggregated demand by fiscal means. According to Buiter (1999), fiscal measures fostering aggregated consumption on national levels can be as effective as a supranational fiscal transfer system. He assumes that fiscal deficits are reflected in current account imbalances. There are indicators for this phenomenon in the EMU as well (see, Sinn and Wollmershäuser 2011). Low price rigidities or, in other terms, low menu costs can also contribute to the absorption of asymmetric shocks (see, Buiter 1999). Baldwin and Wyplosz (2019) point out that asymmetric shocks in a monetary union reveal the biggest challenges for an economy that lost its sovereign monetary policy as a tool to absorb these shocks. OCA theory presumes that joining a monetary union is beneficial if the loss of monetary policy that helps to stimulate economic growth by fostering credit demand through lowering policy rates and strengthening international competitiveness by decreasing exchange rates is compensated by increased competitiveness through better price comparability and higher competition intensity in a more integrated market (see, Jager and Hafner 2013). Among others, Pisani-Ferry (2013) and Krugman (2013) claim that the EMU does not fulfill the OCA conditions and, therefore, is supposedly more vulnerable in times of crisis. The sovereign debt crisis nourished this kind of criticism as it apparently proved the EMU’s and especially Greece’s inability to deal with asymmetric shocks. Among others, the OCA theory provided the theoretical background for arguments in favor of Greece leaving the Eurozone as the OCA criteria are not fulfilled. Referring to OCA theory, a common currency area should be characterized by (i) extensive trade relations, (ii) similar business cycles, (iii) high level of labor mobility, as well as (iv) a mechanism to cushion economic slump, which usually are fiscal transfers (see, Frankel and Rose 1998) in order to address asymmetric shocks.

Among others Fidrmuc (2004), de Grauwe and Mongelli (2005) support an optimistic view regarding the question whether a currency union shortly after having been founded meets the OCA criteria or not. They claim that even though a monetary union might not comply with the conditions of an OCA right away, there are indications that economic integration fosters symmetry in business cycles (see, Rose 2008), whereby a monetary union might comply with OCA criteria over time. According to Frankel and Rose (1998) as well as Mongelli (2002), this phenomenon reflects the endogeneity of OCA, which is to say that a monetary union approaches the OCA criteria through its higher degree of integration which is a result of eliminated exchange rates and increased price transparency. The symmetry in business cycles relieves the members of the monetary union of the disadvantages associated with having abandoned their sovereign monetary policy: in contrast to asymmetric shocks, symmetric economic shocks can also be contained by a common monetary policy. Referring to Buiter (1999), fiscal policy responses are on a national level as effective as on a supranational level. From these perspectives, the question whether the EMU a few years after its introduction has complied with the OCA criteria or not becomes irrelevant over time because a currency union will converge to an OCA.

**Fig. 3:**
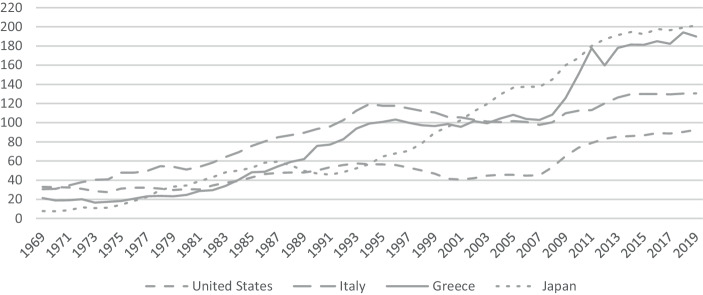
Central government debt to GDP ratio in percentages. Source: IMF (2021)

The exit argument was strengthened further by Reinhart and Rogoff (2010) and later Reinhart et al. (2012), who scrutinized public debt overhangs in advanced economies since the early 1800s. The paper fostered discussions in the economic and political sphere concerning the question of which debt-to-GDP level can still be regarded as sustainable. This would possibly have implications for the Greek rescue program. Reinhart et al. (2012) proclaim that a debt-to-GDP ratio of over 90% is considered threatening to the debt sustainability of developed economies. But in this explanation, not only the structure of the public debt was disregarded but also the question of why, e.g., Japan’s sovereign debt level was considered to be sustainable. In contrast, Greece and other high-debt European countries were deemed to be overindebted even at comparably far lower debt levels (see also, Fig. 3). Instead of associating fiscal sustainability with certain debt levels, the market-oriented response would be that a sovereign’s debt level can be regarded as sustainable as long as private investors are willing to buy government bonds (see, Collignon 2012). The most popular theory of public debt sustainability is based on the idea of an intertemporal budget constraint. It claims that the discounted value of a sovereign’s current and future income has to be sufficient to cover the discounted value of a government’s current and future expenses in addition to the given debt (see, Quintos 1995). Uncertainty might also play a role. In fact, Kotlikoff (2006) has argued convincingly that there are two types of relevant uncertainties: first of all, there is some uncertainty about the economy’s underlying technology and preferences. Secondly, there also is uncertainty about future government policies. In this context, one should be aware of how the interaction between fiscal and monetary policy contributes to fiscal solidity. Public debt rises as long as the real interest rate exceeds the economy’s growth rate and cannot be compensated by the primary surplus. The interdependency of the parameters is well described by the Fisher effect claiming that the real interest rate depends on the nominal interest rate and the inflation rate, which are, together with the growth rate, affected by monetary and fiscal policy (see, Summers 1982). A restrictive fiscal policy is supposed to weaken money demand and thereby put pressure on the interest and inflation rate but it might also be a burden on economic growth (see, Collignon 2012). For more than 200 years, the US could generate growth rates that mostly outran average interest rates (see, Bohn 1998), which did not prevent debt levels from rising (see, Fig. 3).

When the European sovereign debt crisis began to spread in 2010, Japan’s debt-to-GDP ratio already amounted to 198%, while Greece suffered much more from its debt-to-GDP level of then 129% (see, Doi et al. 2011). Additionally, Greece’s debt-to-GDP ratio already amounted to 94.4% in 1993 (see, Gourinchas et al. 2017). Nikiforos et al. (2015) state that shortly before the start of the sovereign debt crisis, 80% of Greek’s sovereign debt was issued in bonds, of which 73% were held by foreign investors, most of which were private financial institutions. This fact supposedly increases the likelihood that investors will withdraw their funds in the event of a crisis. In 2015, the share of foreign creditors remained nearly the same (75%) but had changed from the private to the public sector (see, Nikiforos et al. 2015). In contrast, in Japan, significant parts of the government debt are owned by social security funds which control assets amounting to about 40% of the Japanese GDP (see, Doi et al. 2011).

## Data and methodology

We examine 10-year government bond yields in five different EMU member countries (namely France, Germany, Greece, Italy, and Spain). This monthly data is taken from the Federal Reserve Bank of St. Louis database FRED and was compiled by the Organisation for Economic Co-operation and Development (OECD). More specifically, we examine interest rate differentials (id) of the bonds issued by the other countries relative to German bonds, with x denoting the member states France, Greece, Italy, and Spain, respectively (see, Eq. 1):

1 $$id_{X}=i_{X}-i_{\text{Germany}}$$

Given that the default risk of the Federal Republic of Germany usually is considered to be negligible and that there is a highly liquid and very active market for German government bonds, these debt obligations can be described with the expression of a risk-free asset. Consequently, German sovereign bond yields are frequently used as the EMU benchmark interest rate (see, for example, Gruppe and Lange 2014 and Rodriguez Gonzalez et al. 2017). Therefore, the term id in Eq. 1 can be interpreted as a risk premium. Our data sample covers the time from 2009/10 to 2017/12. In this period, Greek bond yields were particularly high. Thus, we examine data from a phase in which the difficulties in this country should have mattered most for the entire EMU government bond market. Consequently, our empirical work presented below is also an indirect test of the idea that Greece, at least for some time, was of special importance when trying to explain the European sovereign debt crisis (see, most importantly, Arghyrou and Kontonikas 2012). Finding no information flow from the market for Greek bonds to the one for other EMU government bonds even in this period of time would certainly contradict this point of view.

As already noted, we follow Gunay (2020) and use Granger causality tests to improve our understanding of the information flow between different risk premia. This technique has also been employed to test the so-called German Dominance Hypothesis (GDH), which was an important theoretical concept examining European interest rates before the introduction of the common currency (see, for example, Bajo-Rubio and Montávez-Garcés 2002 and Booth and Ciner 2005). The GDH predicts that German interest rates were of special importance for the bond yields in the other member countries of the European Monetary System (EMS) (see, amongst others, Camarero and Tamarit 1995 and Chen and Mazumdar 1995). More specifically, we use procedures developed by Toda and Yamamoto (1995) to test for Granger causality. Concerning the empirical evidence that is reported later in this paper, the empirical study by Booth and Ciner (2005) is of special importance because these authors used a test procedure (which was developed by Dolado and Lütkepohl 1996) that is very similar to the Toda and Yamamoto (1995) test that is employed here. At this point, it has to be noted that Granger causality implies that one time series can be helpful in forecasting a second variable (see, most importantly, Granger 1969). Thus, one time series *X*_*t*_ is said not to be Granger causing the variable *Y*_*t*_ for all $n> 0$ under the condition that:

2 $$F(Y_{t+n}\textbar \Omega _{t})=F(Y_{t+n}\textbar \Omega _{t}-X_{t})$$

In Eq. 2, *F* denotes the conditional distribution and Ω_*t*_is all information that could be of relevance in the context under investigation. There may also be feedback effects between the variables. In this case, bidirectional Granger causality would be present among the variables examined (see, for instance, Patev et al. 2006 and Tholl et al. 2021). Otherwise, there is uni-directional Granger causality. The procedure developed by Toda and Yamamoto (1995) is based on the concept of vector autoregressive models introduced by Sims (1980).

Following Sims (1980) *Y*_*t*_ in Eq. 3 is a vector of $(n\times 1)$ endogenous variables, *A*_*i*_ are $(n\times n)$ coefficient matrices, *C* is a $(n\times 1)$ vector of constants, and *ε*_*t*_ is an $(n\times 1)$ vector of disturbance terms:

3 $$Y_{t}=C+A_{1}Y_{t-1}+A_{2}Y_{t-2}+\cdot \cdot \cdot +A_{p}Y_{t-p}+\varepsilon _{t}$$

In order to test for Granger causality, Toda and Yamamoto (1995) suggested estimating a vector autoregression in levels considering p time lags and extending this model by m time lags (see, Eq. 4). Here m is the highest order of integration of any exogenous variable examined and p is the optimal number of time lags for the vector autoregressive model:

4 $$Y_{t}=C+A_{1}Y_{t-1}+A_{2}Y_{t-2}+\cdot \cdot \cdot +A_{p}Y_{t-p}+\cdot \cdot \cdot +A_{p+m}Y_{t-\left(p+m\right)}+\varepsilon _{t}$$

Using this approach—which often is called a modified Wald test—ensures that the test statistic is asymptotically chi-square distributed by adding m lags in Eq. 4. The optimal number of time lags for the vector autoregression can be selected using the traditional information criteria (here, the AIC) and m is determined employing unit root tests.

## Empirical evidence

To determine the order of integration of the variables under observation here, we use the test suggested by Phillips and Perron (1988) with the critical values tabulated by MacKinnon (1996). The results of these tests examining the four interest rate differentials are presented in Tables 1, 2, 3 and 4.

**Table 1:** Unit Root Test Spread France to Germany

Null Hypothesis: idFrance has a unit root			
Exogenous: Constant			
*Bandwidth: 2 (Newey-West automatic) using Bartlett kernel*			
		Adj. t‑Stat	Prob.*
Phillips-Perron test statistic		−2.018381	0.2786
Test critical values:	1% level	−3.497727	
	5% level	−2.890926	
	10% level	−2.582514	
Null Hypothesis: D(idFrance) has a unit root			
Exogenous: Constant			
*Bandwidth: 0 (Newey-West automatic) using Bartlett kernel*			
		Adj. t‑Stat	Prob.*
Phillips-Perron test statistic		−9.628260	0.0000
Test critical values:	1% level	−3.497727	
	5% level	−2.890926	
	10% level	−2.582514	

*MacKinnon (1996) one-sided *p*-values

**Table 2:** Unit Root Test Spread Greece to Germany

Null Hypothesis: idGreece has a unit root				
Exogenous: Constant				
*Bandwidth: 1 (Newey-West automatic) using Bartlett kernel*				
			Adj. t‑Stat	Prob.*
Phillips-Perron test statistic			−1.884948	0.3381
Test critical values:	1% level		−3.497727	
	5% level		−2.890926	
	10% level		−2.582514	
Null Hypothesis: D(idGreece) has a unit root				
Exogenous: Constant				
*Bandwidth: 5 (Newey-West automatic) using Bartlett kernel*				
			Adj. t‑Stat	Prob.*
Phillips-Perron test statistic			−8.801587	0.0000
Test critical values:	1% level		−3.497727	
	5% level		−2.890926	
	10% level		−2.582514	

*MacKinnon (1996) one-sided *p*-values

**Table 3:** Unit Root Test Spread Italy to Germany

Null Hypothesis: idItaly has a unit root			
Exogenous: Constant			
*Bandwidth: 1 (Newey-West automatic) using Bartlett kernel*			
		Adj. t–Stat	Prob.*
Phillips-Perron test statistic		−1.647776	0.4545
Test critical values:	1% level	−3.497727	
	5% level	−2.890926	
	10% level	−2.582514	
Null Hypothesis: D(idItaly) has a unit root			
Exogenous: Constant			
*Bandwidth: 3 (Newey-West automatic) using Bartlett kernel*			
		Adj. t‑Stat	Prob.*
Phillips-Perron test statistic		−8.014487	0.0000
Test critical values:	1% level	−3.497727	
	5% level	−2.890926	
	10% level	−2.582514	

*MacKinnon (1996) one-sided *p*-values

**Table 4:** Unit Root Test Spread Spain to Germany

Null Hypothesis: idSpain has a unit root			
Exogenous: Constant			
*Bandwidth: 0 (Newey-West automatic) using Bartlett kernel*			
		Adj. t‑Stat	Prob.*
Phillips-Perron test statistic		−1.383385	0.5875
Test critical values:	1% level	−3.497727	
	5% level	−2.890926	
	10% level	−2.582514	
Null Hypothesis: D(idSpain) has a unit root			
Exogenous: Constant			
*Bandwidth: 3 (Newey-West automatic) using Bartlett kernel*			
		Adj. t‑Stat	Prob.*
Phillips-Perron test statistic		−8.531971	0.0000
Test critical values:	1% level	−3.497727	
	5% level	−2.890926	
	10% level	−2.582514	

*MacKinnon (1996) one-sided *p*-values

According to the tests, all four interest rate differentials seem to be non-stationary variables that are integrated of order one. Therefore, when employing the procedure developed by Toda and Yamamoto (1995), m in all cases under investigation here is 1. Given the research question of our empirical study (namely, the role of Greece in the European sovereign debt crisis), we examine the relationship between markets for government bonds from Greece and France, Greece and Italy, as well as Greece and Spain. As already noted, we follow Gunay (2020) and are interested in the information flow between these fixed-income markets. More specifically, we examine the risk premia. Therefore, we analyze interest rate differentials relative to German 10-year government bond yields and estimate three augmented vector-autoregressive models (as suggested by Toda and Yamamoto 1995) to test for Granger causality. The results are reported in Table 5, 6 and 7. As already discussed, m in all cases is 1 because all the time series examined are integrated of order one. The information criterium AIC is used to determine p and results in p being 5 with regard to France, 6 with regard to Italy, and 7 with regard to Spain.

**Table 5:** Granger Causality Test examining France

TY Granger Causality Tests			
Sample: 2009M10 2017M12			
Included observations: 99			
*Dependent variable: idFrance*			
Excluded	Chi-sq	df	Prob
idGreece	11.12782	5	0.0489
*Dependent variable: idGreece*			
Excluded	Chi-sq	df	Prob
idFrance	54.12560	5	0.0000

**Table 6:** Granger Causality Test examining Italy

TY Granger Causality Tests			
Sample: 2009M10 2017M12			
Included observations: 99			
*Dependent variable: idItaly*			
Excluded	Chi-sq	df	Prob
idGreece	26.77646	6	0.0002
*Dependent variable: idGreece*			
Excluded	Chi-sq	df	Prob
idItaly	27.63546	6	0.0001

**Table 7:** Granger Causality Test examining Spain

TY Granger Causality Test			
Sample: 2009M10 2017M12			
Included observations: 99			
*Dependent variable: idSpain*			
Excluded	Chi-sq	df	Prob
idGreece	20.63153	7	0.0044
*Dependent variable: idGreece*			
Excluded	Chi-sq	df	Prob
idSpain	31.51690	7	0.0000

In all cases, the hypothesis of no Granger causality can be rejected at the 5% error level. Therefore, all three models indicate that there is bidirectional Granger causality between the examined bond yield spreads. Consequently, Greek interest rate differentials to Germany help to forecast the bond yield spreads of the other three countries (France, Italy, and Spain) and the interest rate differentials to Germany for French, Italian, and Spanish government bonds seem to be helpful to predict the spread between 10-year government bond yields of Greece and Germany. Therefore, data from Greece does matter for the EMU government bond market. This empirical finding is compatible with the ideas highlighted by Arghyrou and Kontonikas (2012). In fact, the data seem to imply that there are good reasons to divide the sovereign debt crisis in Europe into an early (II a) and a later (II b) phase.

## Conclusion

This empirical study examines the European sovereign debt crisis and focuses on the role of Greece. More specifically, we first discuss the question of fiscal stability in Greece and then analyze interest rate differentials to Germany of several EMU member countries (namely France, Greece, Italy, and Spain). We show that bidirectional Granger causality between all examined bond yield spreads does indeed exist. Therefore, the information embedded in Greek government bond yields (risk premia) seems to be helpful in forecasting interest rates in the economically more important member countries France, Italy, and Spain. This empirical finding is of some interest when trying to identify different phases of the crisis. Greece seems to matter in this context (see, most importantly, Arghyrou and Kontonikas 2012). Our results reported above are not only interesting from a macro-economic point of view. In fact, fixed-income investors and risk managers working for financial institutions with high exposures to sovereign bonds issued by EMU member countries (e.g., European insurance companies) should also pay attention to this aspect. Our findings might, for example, be of relevance for models of government bond yield spreads in the monetary union. Given that Lempérière et al. (2017) highlighted that there still is no absolutely clear picture of how financial markets determine risk premia, the results reported in this paper clearly could be of value for investors and risk managers controlling asset managers when investing in government bonds or related assets.
